# Structural chemistry-guided revelation of superior thermally insulative TeI_4_

**DOI:** 10.1093/nsr/nwaf544

**Published:** 2025-12-02

**Authors:** Qingyu Bai, Zhiwei Chen, Ziyue Liu, Linjie Wu, Changyuan Li, Jiong Yang, Jun Luo

**Affiliations:** Interdisciplinary Materials Research Center, School of Materials Science and Engineering, Tongji University, Shanghai 201804, China; Interdisciplinary Materials Research Center, School of Materials Science and Engineering, Tongji University, Shanghai 201804, China; Interdisciplinary Materials Research Center, School of Materials Science and Engineering, Tongji University, Shanghai 201804, China; Interdisciplinary Materials Research Center, School of Materials Science and Engineering, Tongji University, Shanghai 201804, China; Interdisciplinary Materials Research Center, School of Materials Science and Engineering, Tongji University, Shanghai 201804, China; Materials Genome Institute, Shanghai Engineering Research Center for Integrated Circuits and Advanced Display Materials, Shanghai University, Shanghai 200444, China; Interdisciplinary Materials Research Center, School of Materials Science and Engineering, Tongji University, Shanghai 201804, China

**Keywords:** thermal insulation materials, structural chemistry, phonon engineering, iodides

## Abstract

As the cornerstone of structural chemistry, the elemental compositions and spatial arrangements of atoms determine the functionalities of compounds. This principle is fully epitomized by ‘magic’ angle materials, where the lattice twisting extends the periodicity of the Moiré superlattice, revealing many unexpected properties. Here, we investigate how the extended lattice periodicity affects the properties of lattice dynamics, with a primary focus on thermal conductivity. Through the modulation of bond length and angle, the lattice periodicities of binary iodides CsI, BaI_2_, BiI_3_ and TeI_4_ are extended as the cationic valences increase from monovalent to tetravalent states, leading to a substantial decrease in thermal conductivity. It is revealed that even in a simple binary compound like TeI_4_, an extremely low thermal conductivity of 0.17 W m^−1^ K^−1^ at room temperature can be achieved. Compared to CsI, BaI_2_ and BiI_3_, the superior heat insulation of TeI_4_ is found to stem from the large extended periodicity of the atomic arrangement enabled by having nearly an order of magnitude more atoms in the primitive cell.

## Teaser

By manipulating the cation valences in binary iodides, this work achieves an extremely low thermal conductivity in TeI4, revealing how extended lattice periodicity lead to superior thermal insulation.

## INTRODUCTION

Thermal conductivity serves as a key metric for evaluating performance of materials for thermal management [1], with critical applications in thermoelectrics [2], energy-efficient buildings [3] and aerospace [4]. Given that most of these applications additionally require mechanical robustness, solid-state materials are prioritized in the practical applications, in which electrons and phonons dominate the heat transport of materials. The advanced heat insulators are usually non-metals because the conduction of electrons could largely contribute to heat conduction [5]. Therefore, the manipulation of phonon transport is crucial for advancements in heat insulation [6].

As part of lattice dynamics, the generation, propagation and scattering of phonons originate from the vibrational behavior of atoms at equilibrium positions, governed by the periodic potential fields of lattice. These characteristics of structural chemistry are intrinsically determined by the specific chemical environment surrounding each atom or ion, including the elemental compositions, interatomic distances, coordination numbers and spatial arrangement of neighboring atoms. It is theoretically suggested that the large coordination number in a polyhedron and certain local interactions would increase the delocalization of bonding electrons and induce the asymmetric long-range interaction of periodic potential field, thereby increasing the phonon anharmonicity [7]. This is supported by computational and experimental evidence, where the resonant bonding [8] and metavalent bonding [9–11] in IV–VI compounds lead to low lattice thermal conductivity (*κ*_L_).

The periodicity of potential fields would be broken in the presence of local lattice twisting, stacking or distortions, which further increases the complexity of the coordination environment and extends the atomic arrangement periodicity. For examples, in MoS_2_ Moiré superlattices [12], tuning the interlayer twist angle markedly enhances the phonon anharmonicity through controlled modification of periodic potential fields; the strong phonon localization in two-angle disordered twisted multilayer graphene [13] leads to a giant *κ*_L_ reduction. For an extreme case, amorphous materials achieve complete non-periodicity, a state exhibiting short-range order while lacking long-range periodicity, in which the extremely low *κ*_L_ is usually observed in such materials [14,15].

To understand the underlying mechanisms by which the extended periodicity affects *κ*_L_, it is necessary to further evaluate the three key parameters for the phonon transport, namely specific heat (*C*_v_), sound velocity (*v*) and relaxation time (*τ*) [16]. Since *C*_v_ saturates at or above room temperature, *v* and *τ* are primarily focused on. The former can be expressed as:


(1) \begin{eqnarray*}
v = \frac{{\mathop \sum \nolimits_{i = 1}^n {r}_i}}{n}\sqrt {\frac{{{{\left( {\mathop \prod \nolimits_{i = 1}^n {f}_i} \right)}}^{2/n}}}{{\frac{{\mathop \sum \nolimits_{i = 1}^n {f}_i}}{n}\frac{{\mathop \sum \nolimits_{i = 1}^n {m}_i}}{n}}}},
\end{eqnarray*}

where *n* is the number of atoms in the primitive cell, *r_i_* is the *i*th bond length, *f_i_* is the *i*th force constant between the nearest neighbors, and *m_i_* is the mass of *i*th atom [17]. The extended periodicity is typified by amorphization with extremely large *n* for forming local supercells, because the disorder leads to some bonds elongating and some shortening. Accordingly, some force constants will decrease and some will increase, leading to the so-called force constant fluctuation. Under the perturbation theory, the mathematical expectation of the force constant (the arithmetic mean, Σ*f_i_*/*n* = *f*_0_) remains unchanged. In the simplest case, if the local supercell after amorphization is twice the size of the primitive cell, one *f*_0_ due to fluctuation can decrease to 0.5*f*_0_ and another *f*_0_ increase to 1.5*f*_0_, for example. According to Equation 1, the geometric mean for the supercell, (Π*f_i_*)^2/^*^n^* = (0.5*f*_0_*1.5*f*_0_)^2/2^, is less than that for the primitive cell, (Π*f_i_*)^2/^*^n^* = *f*_0_^2/1^. This helps us understand the reduction in *v* due to the extend periodicity of the lattice [18], where the fluctuations of force constants correct the quasi-elastic properties of lattice dynamics. For the inelastic properties in lattice dynamics, *τ* is primarily determined by the phonon anharmonicity [19], which is also related to the chemical environment, including bond coordination [7], bond characteristics [8,20] and bond anisotropy [21,22].

It is an effective strategy to extend the lattice periodicity by changing the atomic valence states in transition metal oxides [23,24]. In manganese oxides, the multiple-valence-states Mn ions adopt distinct coordination geometries with oxygen ligands, forming compounds such as MnO, Mn_2_O_3_, Mn_3_O_4_ and MnO_2_ [23]. With the addition of variations in ligand connectivity, the complexity of the coordination environments substantially increases, resulting in a nearly 15 times greater expansion of the primitive cell volume. Similarly, for Cs_2_Ge_3_Ga_6_Se_14_ with both Ge^3+^ and Ge^2+^ ions, the structure features [Ge(1)^3+^_2_Se_6_] dimers and [Ge(2)^2+^Se_6_] octahedra, which reduces the lattice periodicity due to the rich diversity in coordination environments [25].

For designing the heat insulators, this work is motivated by these linkages between structural chemistry and heat conduction behavior, mainly involving CsI, BaI_2_, BiI_3_ and TeI_4_ binaries due to their simple compositions. Additionally, these compounds exhibit a comparably large average atomic mass (135 ± 10% g mol^−1^) and a broad range of cation valence states (+1 to +4), which are expected to enrich the structural chemistry characteristics. It is revealed that BiI_3_ possesses strong anharmonicity and low *v*, resulting in a low *κ* of 0.3 W m^−1^ K^−1^ at 300 K. Interestingly, the extended atomic arrangement periodicity induced by nearly an order of magnitude more atoms in the primitive cell of TeI_4_, results in an extremely low thermal conductivity of 0.17 W m^−1^ K^−1^ at 300 K compared to the confirmed literature minimum of ∼0.2 W m^−1^ K^−1^ to date for dense solids [26–29].

## RESULTS AND DISCUSSION

**Figure 1.**
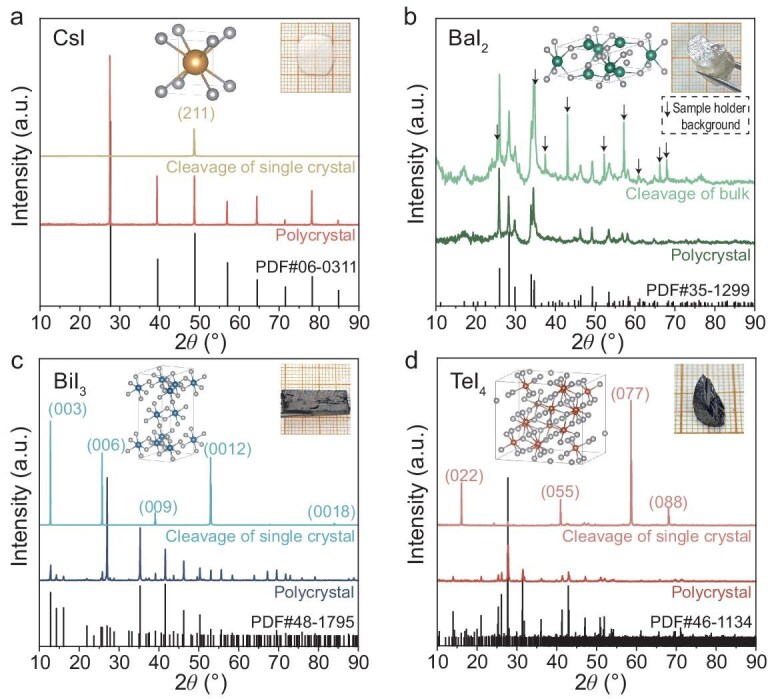
Crystal structures. Room-temperature XRD patterns for CsI (a), BaI_2_ (b), BiI_3_ (c) and TeI_4_ (d). Inset: the crystal structures and typical photographs of crystals.

Details about synthesis, characterization and measurement are provided in the Supplementary data. X-ray diffraction (XRD) results (Fig. 1 and Fig. S1) confirm the crystal structures of CsI, BaI_2_, BiI_3_ and TeI_4_ crystals, which are consistent with literature reports [30–33]. The increase in cation valence across these four compounds results in distinct lattice structure symmetries: *Pm*-3*m* for CsI, *P*-62*m* for BaI_2_, *R*-3 for BiI_3_ and *Pnma* for TeI_4_. Moreover, the primitive cell volume increases by over one order of magnitude from CsI to TeI_4_ (Table S1). The single crystals of these compounds were grown to investigate the intrinsic thermal transport properties. The cleavage planes corresponding to the [211], [001] and [011] crystallographic orientations were observed in CsI, BiI_3_ and TeI_4_ crystals, respectively (Fig. 1). For a consistent comparison of thermal transport along the same direction, a single crystal with the (001) crystallographic plane of CsI was obtained (Fig. S2). Due to the unavailability of oriented single crystals, the XRD patterns of BaI_2_ were collected from polycrystalline ingot fracture surfaces (Fig. 1). The pattern exhibits background peaks originating from the sample holder, with the corresponding peak listed in Fig. S3. The elemental homogeneity is further verified by scanning electron microscopy and energy dispersive spectroscopy mapping (Fig. S4). All the pellet samples obtained by hot pressing achieve >95% density. The details of the hot-pressing parameters compared with the crystallographic parameters of these four compounds at 300 K are provided in Tables S1 and S2.

**Figure 2.**
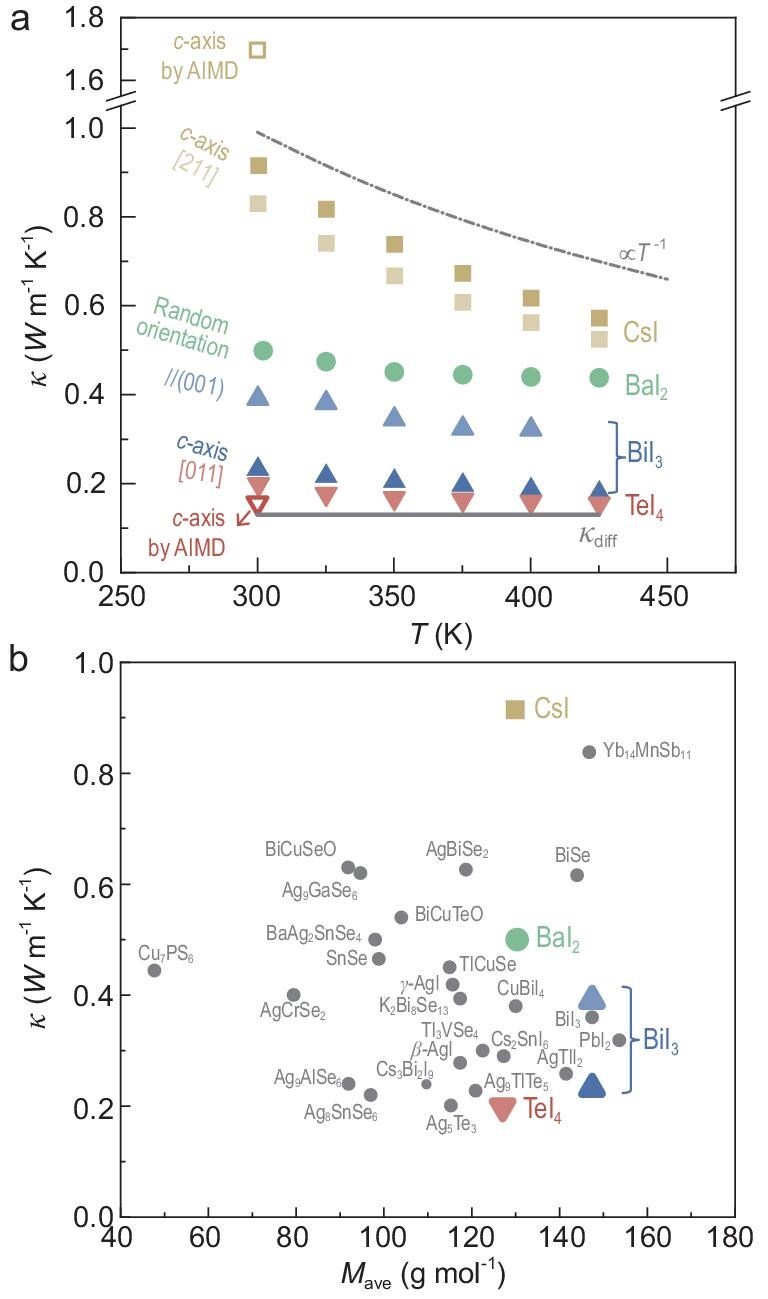
Thermal transport properties. (a) Temperature-dependent total thermal conductivity (*κ*) for CsI, BaI_2_, BiI_3_ and TeI_4_ crystals along different crystallographic directions. Predictions based on AIMD and the diffuson model [15] are included. (b) Room-temperature *κ* as a function of average atomic mass (*M*_ave_), for CsI, BaI_2_, BiI_3_ and TeI_4_ and materials reported in the literature [17,18,28,29,37–55].

The total thermal conductivity (*κ*) in CsI, BaI_2_, BiI_3_ and TeI_4_ is primarily attributed to the lattice *κ* (*κ*_L_) due to their extremely low electrical conductivity (Fig. S5). In order to have a consistent comparison on thermal conductivity between this work and the literature, *κ* is estimated by the Dulong–Petit heat capacity (Fig. S6). With an increase in cation valence, the gradual decreases in *κ* are observed for the CsI, BaI_2_, BiI_3_ and TeI_4_ crystals (Fig. 2a), as well as for the hot-pressed pellets (Fig. S7). Overall, the *κ* of crystals consistently exceeds that of pellets, primarily due to reduced grain boundary and defect scattering. Notably, BiI_3_ exhibits pronounced anisotropy in *κ* between the in-plane and out-of-plane directions.

*Ab initio* molecular dynamics (AIMD) simulations yielded a *c*-axis thermal conductivity for CsI of ∼1.7 W m^−1^ K^−1^ at 300 K, which falls within the acceptable uncertainty range typical for the first-principles calculations. For TeI_4_, the computed *c*-axis thermal conductivity is slightly lower than the experimentally measured value along the [011] direction. The AIMD-computed thermal conductivities along the *a*-, *b*- and *c*-axes for both CsI and TeI_4_ at 300 K are summarized in Table S3. CsI exhibits isotropic thermal conductivity, while TeI_4_ shows weak anisotropy among the three crystallographic directions.

More interestingly, as the valence states of cations increase, the temperature dependence of thermal conductivity weakens. The nearly 1/*T* dependent thermal conductivity for CsI suggests the Umklapp phonon scattering dominance. In contrast, the weak temperature dependence observed in TeI_4_ might originate from possible mechanisms, such as wave-like-dominant two-channel transport [28,29,34,35] and phonon renormalization [36]. Considering the burden of extremely lager computations, such deviation of 1/*T* dependence deserve a further investigation. Compared to the literature results on *κ* for heat insulators in dense form [17,18,28,29,37–55], TeI_4_ shows a superior heat insulation (Fig. 2b). All four binaries with *κ* of <1 W m^−1^ K^−1^ share large average atomic mass (*M*_ave_) as a unifying feature.

**Figure 3.**
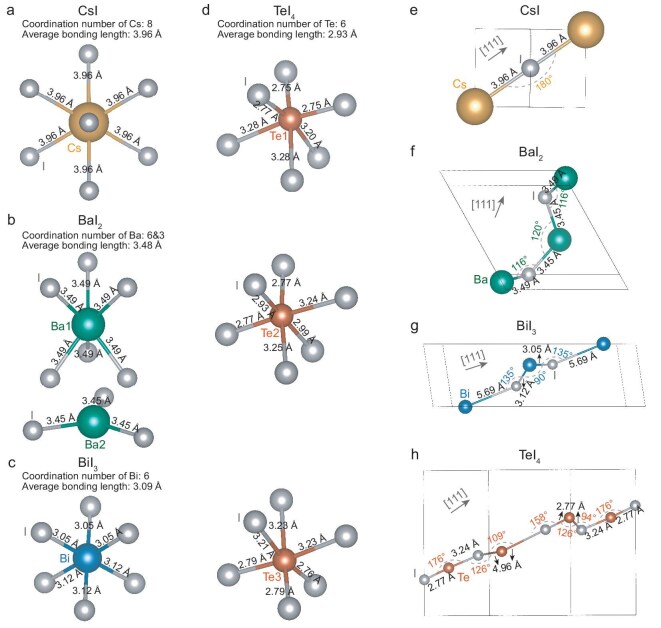
Chemical bond environment. Bonds centered at cations (a–d) and atomic arrangement periodicity along the [111] direction (e–h) for CsI (a and e), BaI_2_ (b and f), BiI_3_ (c and g) and TeI_4_ (d and h).

With cations as the coordination centers, the local atomic structures of all four compounds exhibit large coordination numbers (*CN* ≥ 6) (Fig. 3a–d). Such high *CN* values are commonly associated with low *κ*_L_, because the electron delocalization would increase the lattice anharmonicity due to the distortion of potential function [7]. It can be found that TeI_4_ displays the most complex cationic chemical environment, as evidenced by variations in both bond lengths and bond angles (Fig. 3d). The extended periodicity of chemical bonds is shown along the [111] direction (Fig. 3e–h), revealing the 3D atomic arrangements. It can be found that TeI_4_ exhibits the longest periodicity of chemical bond, characterized by maximal atomic counts and widest bond length/angle distributions. All these structural features collectively influence the anharmonicity and *v* [20,22].

**Figure 4.**
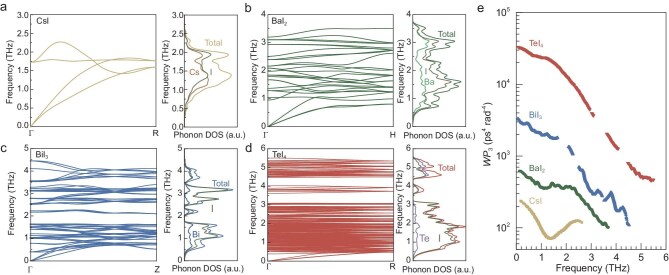
Phonon behavior. DFT-calculated phonon spectra along high-symmetry pathways and pDOS for CsI (a), BaI_2_ (b) BiI_3_ (c) and TeI_4_ (d). (e) Calculated WP_3_ at 300 K.

The impact of extended lattice periodicity on phonon transport is investigated, based on the density functional theory (DFT) calculations for phonon dispersion spectra of CsI, BaI_2_, BiI_3_ and TeI_4_ (Fig. S8a–S8d). To better reflect the 3D characteristics, the high-symmetry pathways from Γ to R for CsI, Γ to H for BaI_2_, Γ to Z for BiI_3_, and Γ to R for TeI_4_ are focused (Fig. 4a–d). The increase in the number of optical branches arises from the expansion of the primitive cell volume induced by extended lattice periodicity. This leads to an increased proportion of optical heat capacity within the total heat capacity, thus reducing the contribution of acoustic heat capacity to the total thermal conductivity [6,18].

The phonon density of states (pDOS) derived from the dispersion spectra (Fig. 4a–d) reveals that the contribution of iodine atoms to the overall vibrational modes increases with the extension of lattice periodicity. This trend originates from the changes in the coordination environment induced by increasing the cation valence (Fig. 3). In CsI, both the monovalent Cs and I atoms possess 8-fold coordination with high charge symmetry, resulting in relatively uniform bond strengths; consequently, anions and cations contribute comparably to the vibrational modes. As the cation valence increases to divalent for Ba, trivalent for Bi and tetravalent for Te, the iodine anions experience decreased *CN* to form polyhedra. Consequently, the high-valence cations with strong binding forces are constrained at the polyhedral centers (Fig. S9), while the terminally located iodine anions are more sensitive to thermal vibration.

The folding of the Brillouin zone induced by extended lattice periodicity reduces the frequencies of the first optical branches, of which the frequency decreases from 1.7 THz for CsI to 0.38 THz for TeI_4_ (Fig. 4). The low-frequency optical branch suppresses the slopes of the acoustic branches, i.e. the phonon group velocities of acoustic phonons. More importantly, the scattering phase space for full-frequency phonons is significantly enhanced (Fig. 4e) due to the existence of the low-frequency optical branch. It is demonstrated that the weighted phase space for three-phonon scattering (*WP*_3_) across the entire frequency range is exponentially enhanced from CsI to TeI_4_. This indicates a remarkable increase in three-phonon scattering processes with the extension of lattice periodicity. Consequently, the reduction in group velocity of heat-carrying acoustic phonons and the exponential enhancement in scattering phase space collectively constitute the primary factors driving the suppression of thermal conductivity.

**Figure 5.**
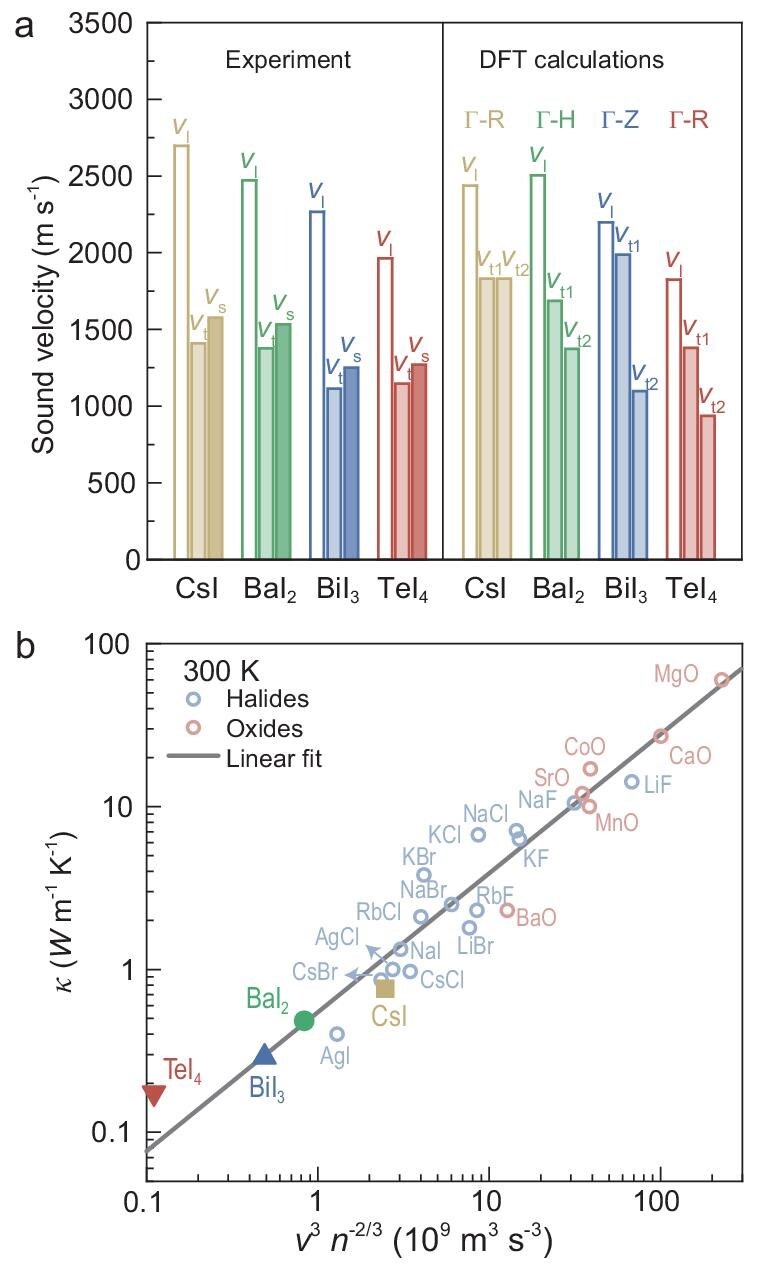
Lattice dynamic properties. Experimental and simulated *v* (a) for CsI, BaI_2_, BiI_3_ and TeI_4_, along with a comparison on thermal conductivity (b) for various binary compounds.

The differences in chemical bond features could also affect *v* (Fig. 5a), according to Equation 1. Figure 3a–d represents the shrinkage of average bond length from CsI (3.96 Å) to TeI_4_ (2.93 Å). As shown in Fig. S9, the strong localization of electron localization functions corresponds to the strong bond ionicity. Conversely, BaI_2_, BiI_3_ and TeI_4_ exhibit more dispersed charge density, indicating the increased covalent bond component. This transition correlates with the increase in the cation electronegativity from Cs to Te. The *v* obtained from experiments using polycrystalline samples and from DFT calculations both reveal that *v* gradually decreases with the extended lattice periodicity. The similar *v* of BiI_3_ and TeI_4_ is primarily the result of the highest mean atomic mass (*M*_ave_ ∼147.5 g mol^−1^) of BiI_3_ among the investigated four compounds. Based on the measured sound velocities and the Leont’ev model [56] for a continuous elastic medium, the lattice dynamic properties such as Debye temperature (*θ*_D_), shear (*G*) and bulk (*B*) modulus, Poisson’s ratio (*σ*) and Grüneisen parameter (*γ*) are estimated and listed in Table S4. It is assumed that acoustic phonons primarily determine the overall thermodynamic properties; the difference between longitudinal *v* (*v_l_*) and transverse *v* (*v_t_*) enables a measure of anharmonicity by the Grüneisen parameter.

According to the Slack [57] and Snyder [58] models, the contribution of acoustic phonons to thermal conductivity is proportional to *τ* and *v*^3^*n*^−2/3^. Here, the *v* term (from 1576^3^ for CsI to 1271^3^ for TeI_4_) contributes a factor of two for thermal conductivity reduction. Based on the AIMD predictions (Fig. 2a), the thermal conductivity decreases by 10 times from CsI to TeI_4_, which suggests that the *τ* term contributes to the rest of the thermal conductivity reduction. Additionally, the large number of atoms in the primitive cell in TeI_4_ is also responsible for the observed low *κ*, as shown in Fig. 5b and Table S5. The low room-temperature *κ* of 0.3 W m^−1^ K^−1^ for BiI_3_ is then clearly attributed to its low *v* with a strong lattice anharmonicity. In contrast, the extremely low thermal conductivity in TeI_4_ is largely due to the large extended periodicity of lattice (Fig. 3h) induced by the large number of atoms in its primitive cell [18], despite the *v* of TeI_4_ being comparable to that of BiI_3_.

## SUMMARY

Taking a series of binary iodides as exemplary models, this work illustrates structural chemistry-guided approaches for advancing heat-insulation applications. This successfully leads to a revelation of superior heat insulation behavior, even in a simple composition of TeI_4_. Focusing on compounds with even stronger bond anisotropy, the extended periodicity of a lattice is therefore believed to be an effective route for further advancements in solid-state heat insulation.

## Supplementary Material

nwaf544_Supplemental_File
