# Supplementary material for: Structural chemistry-guided revelation of superior thermally insulative TeI4
Source: Natl Sci Rev. 2025 Dec 2;13(2):nwaf544. doi: 10.1093/nsr/nwaf544 (PMC12831026; doi:10.1093/nsr/nwaf544)
Supplement: nwaf544_Supplemental_File [file nwaf544_supplemental_file.pdf]

Supplementary

Structural chemistry guided revelation of superior thermally insulative TeI_4

Qingyu Bai[1], Zhiwei Chen[1,*], Ziyue Liu[1], Linjie Wu[1], Changyuan Li[1], Jiong Yang[2,*] and Jun Luo[1,*]

[1]Interdisciplinary Materials Research Center, School of Materials Science and Engineering, Tongji University, Shanghai 201804, China

[2]Materials Genome Institute, Shanghai Engineering Research Center for Integrated Circuits and Advanced Display Materials, Shanghai University, Shanghai 200444, China

***Corresponding authors**. E-mails: 14czw@tongji.edu.cn; jiongy@t.shu.edu.cn; junluo@tongji.edu.cn

Section S1: Synthesis and methods

Synthesis and Microscopic Characterization: High-purity CsI (Aladdin, 99.99%), BaI_2 (Sigma-Aldrich, 99.99%), BiI_3 (Aladdin, 99.99%), and TeI_4 (Aladdin, 99.99%) were used for this study. These materials were hand-ground into fine powders to facilitate the subsequent processing. The powders were subjected to X-ray diffraction (XRD, DX-2700) analysis to characterize their crystalline structures. Subsequently, the powders were compacted into pellet samples using an induction heating hot press system[1], with uniaxial pressure applied according to the parameters detailed in Table S1. The resulting dense samples, exhibiting a density exceeding 95% of the theoretical value, had a diameter of approximately 12 mm and a thickness of around 1.2 mm.

Crystals of CsI, BaI_2, BiI_3 and TeI_4 were grown using the vertical gradient freeze technique. Sealed ampoules containing the respective materials were positioned within a vertical temperature gradient furnace. The ampoule tips were heated to ~30 K above each compound's melting point and held for 2 h, followed by controlled cooling at ~0.7 K h^{-1} to ~50 K below the melting point. Subsequently, the samples were cooled to room temperature at 10-20 K h^{-1}. Crystal specimens prepared for thermal transport measurements exhibited densities >97% of theoretical values, with thicknesses of approximately 1-1.3 mm.

X-ray diffraction measurements were performed on cleavages of the single crystals using a Rigaku SmartLab X-ray diffractometer (Cu-Kα radiation, 60 kV, 150 mA). The Laue diffraction pattern was obtained using a TDF-3000 X-ray crystal analyzer (Dandong Tongda Technology Co., Ltd.). The microstructures of samples were characterized using scanning electron microscopy (SEM, Phenom Pro) coupled with energy-dispersive spectroscopy (EDS).

Measurements: Thermal diffusivity (D) was assessed via laser flash analysis using a Netzsch LFA 467 instrument. The thermal conductivity (κ) was subsequently calculated using the relation $\kappa=\rho C_p D,$ where ρ denotes the density of the pellet, determined from its mass and geometric volume, and C_p represents the heat capacity, which was derived from the Dulong–Petit limit and assumed to be temperature-independent. The use of the Dulong-Petit limit to approximate the specific heat capacity is motivated by two key considerations: (1) the measured specific heats for these four compounds indeed show the average value very close to the Dulong-Petit limit (Figure S6); (2) most of the literatures used specific heat of Dulong-Petit limit for thermal conductivity calculation. This variation of heat capacity primarily results from the inherent conflict between kinetic measurement and thermodynamic equilibrium in DSC. DSC is a dynamic process rather than a fully equilibrium process. The DSC measurement of C_p is essentially measuring the heat flow rate (dQ/dt) as a function of temperature. The three-step method with a standard reference assumes identical sample and reference temperatures, but the practical thermal conductivity differences cause deviations. At slower heating rates, sufficient heat transfer time allows the sensor temperature to closely match the sample's temperature, yielding stable heat flow. In contrast, excessively high heating rates prevent uniform heat distribution, causing the sample temperature to lag behind that of the sensor. At the same time, this requires higher instantaneous power, raising the apparent heat flow and overestimating C_p. However, overly slow heating rates also introduce problems such as baseline drift and enhanced signal-to-noise ratio. Therefore, obtaining highly accurate C_p values remains challenging in such dynamic processes, which is why the Dulong-Petit limit is often adopted as an approximation for C_p in many materials, avoiding errors introduced by varying measurement parameters and facilitating comparison with other studies[2]. Using thermal conductivity derived from a Dulong-Petit limit of C_p is essential for ensuring a consistent and meaningful comparison to literature results.

Specific heat capacity was determined using a NETZSCH DSC 3500 instrument via a three-step measurement procedure, with sapphire as the certified reference material. The sound velocity was determined using an ultrasonic pulse-receiver (Olympus-NDT) coupled with an oscilloscope (Keysight). The ultraviolet-visible spectrum was recorded using a Shimadzu UV-3600 Plus spectrophotometer.

Calculations: The calculations are implemented using the Vienna *Ab Initio* Simulation Package (VASP) based on density functional theory (DFT)[3] with the projector augmented wave (PAW) method[4] and Perdew-Burke-Ernzerhof (PBE) exchange-correlation functional[5]. The energy convergence value between two consecutive steps is set as 5×10^{-8} eV when optimizing atomic positions and the maximum Hellmann-Feynman (HF) force acting on each atom is 5×10^{-5} eV Å^{-1}.

For calculating the phonon properties, a 6×6×6 supercell (432 atoms) of CsI, a 2×2×4 supercell (144 atoms) of BaI_2, a 3×3×1

supercell (216 atoms) of BiI_3 and a 2×2×2 supercell (640 atoms) of TeI_4 were used. The finite-displacement method[6] with a displacement of 0.01 Å was used to calculate the phonon dispersions at 300 K. The weight three-phonon scattering phase space were calculated using home-made code[7] with a *q*-grid of 20×20×20/10×10×18/12×12×4/8×8×8 for CsI/BaI_2/BiI_3/TeI_4.

To obtain the thermal conductivity corresponding to each crystal axis, *ab initio* molecular dynamics (AIMD) simulations at 300 K was carried out, with an energy cutoff of 170 eV and 140 eV for CsI (6×6×6 supercell) and TeI_4 (2×2×2 supercell), respectively. The AIMD time step was set to 1.0 fs, and 22000 steps were conducted. 340 configurations (neglecting the initial 5000 steps, one configuration from 50 steps) were extracted from the AIMD trajectories to fit the force constants through hiphive code[8]. The second-order and third-order force constants were utilized to calculate the lattice thermal conductivities through ShengBTE code[9].

The average sound velocity (v) was estimated from transverse (v_t) and longitudinal (v_l) sound velocities using the following formula,

$$\frac{1}{v^3} = \frac{1}{3}\left(\frac{1}{v_l^3} + \frac{2}{v_t^3}\right) \tag{S1}$$

Likewise, the other physical parameters related to the sound velocity were subsequently determined using the following relationships (S2-S6). The Debye temperature (θ_D) was determined using the formula,

$$\theta_D = \frac{\hbar}{k_B}\left(\frac{6\pi^2}{V}\right)^{\frac{1}{3}} v \tag{S2}$$

where V is the average atomic volume.

The shear modulus (G) and the bulk modulus (B) was determined by the following formula,

$$G = v_t^2 \rho \tag{S3}$$

$$B = v_l^2 \rho - \frac{4}{3} G \tag{S4}$$

where ρ is the theoretical density of compound.

The Poisson's ratio (σ) and the Grüneisen parameter (γ) are calculated by the following formula,

$$\sigma = \frac{\left(\frac{v_l}{v_t}\right)^2 - 2}{2*\left(\frac{v_l}{v_t}\right)^2 - 2} \tag{S5}$$

$$\gamma = \frac{3}{2}\left(\frac{3v_l^2 - 4v_t^2}{v_l^2 + 2v_t^2}\right) \tag{S6}$$

Section S2: Synthesis, phonon behavior and thermal properties

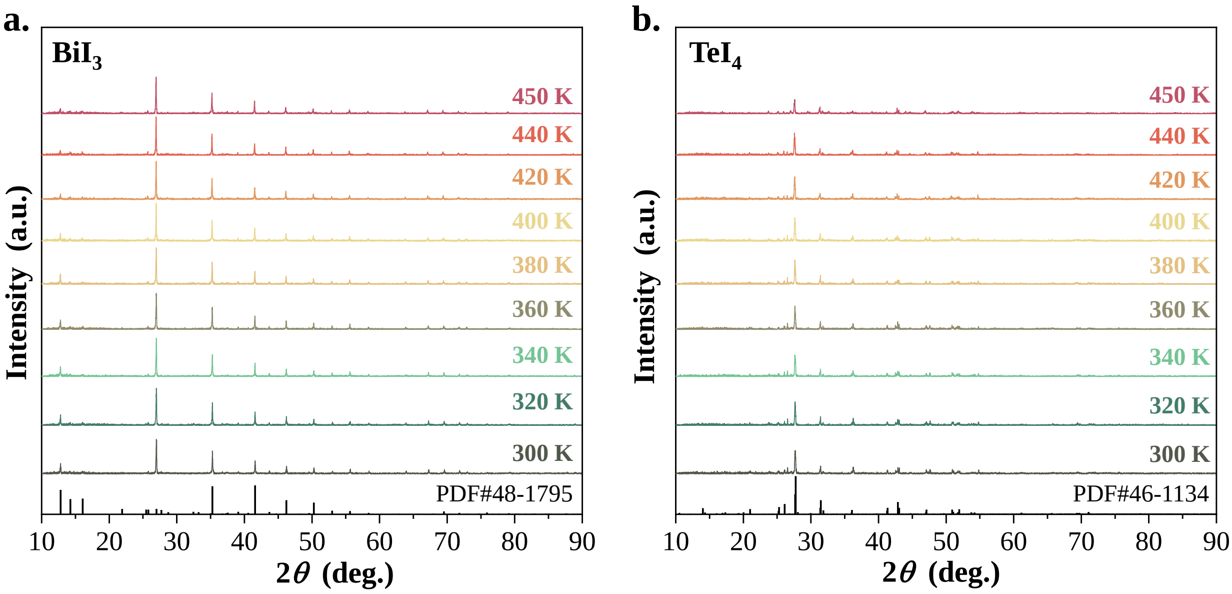

Figure S1. Temperature dependent XRD patterns of BiI_3 (a) and TeI_4 (b) from 300 K to 450 K.

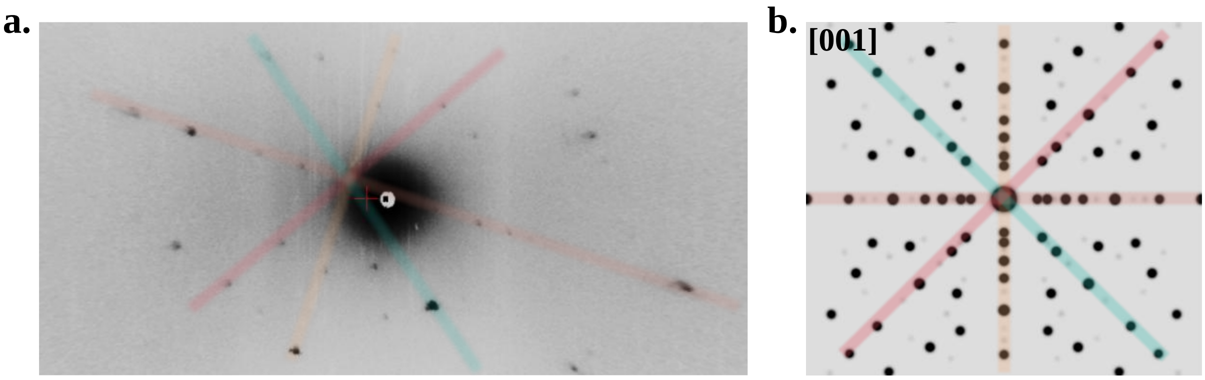

Figure S2. Experimental Laue diffraction patterns (a) and simulated patterns (b) along the [001] direction of CsI.

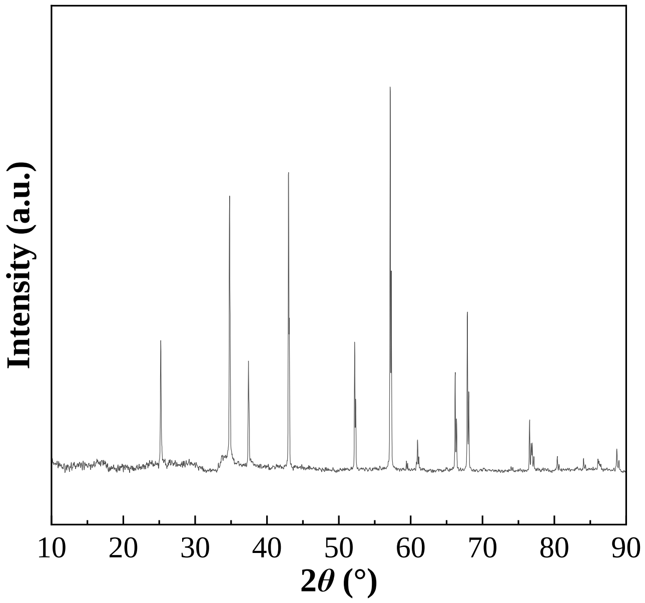

Figure S3. Reference XRD pattern of the sample holder at 300 K.

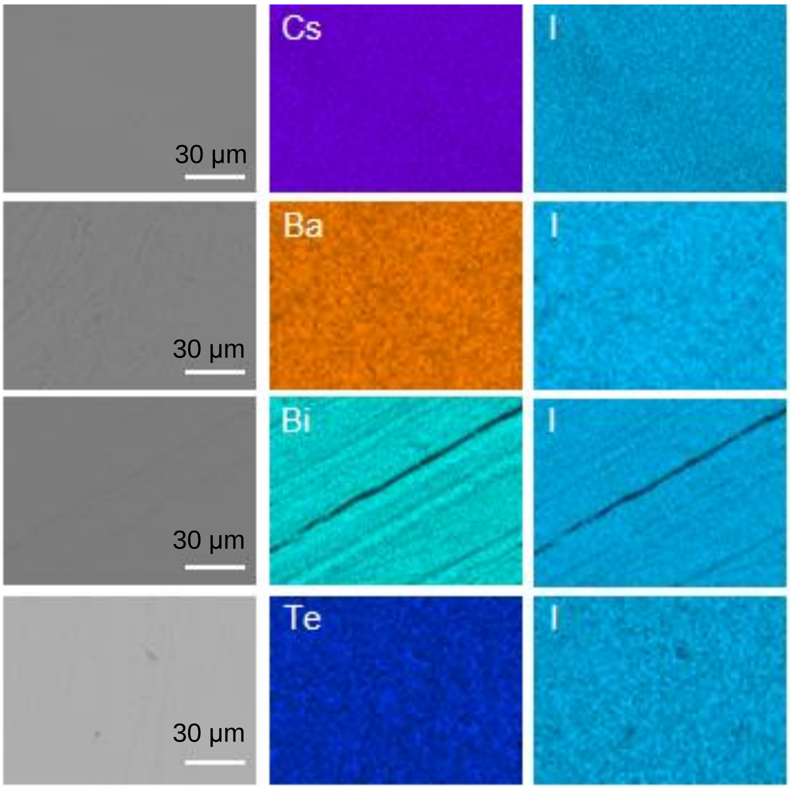

Figure S4. SEM images and the corresponding EDS mapping for CsI, BaI2, BiI3 and TeI4.

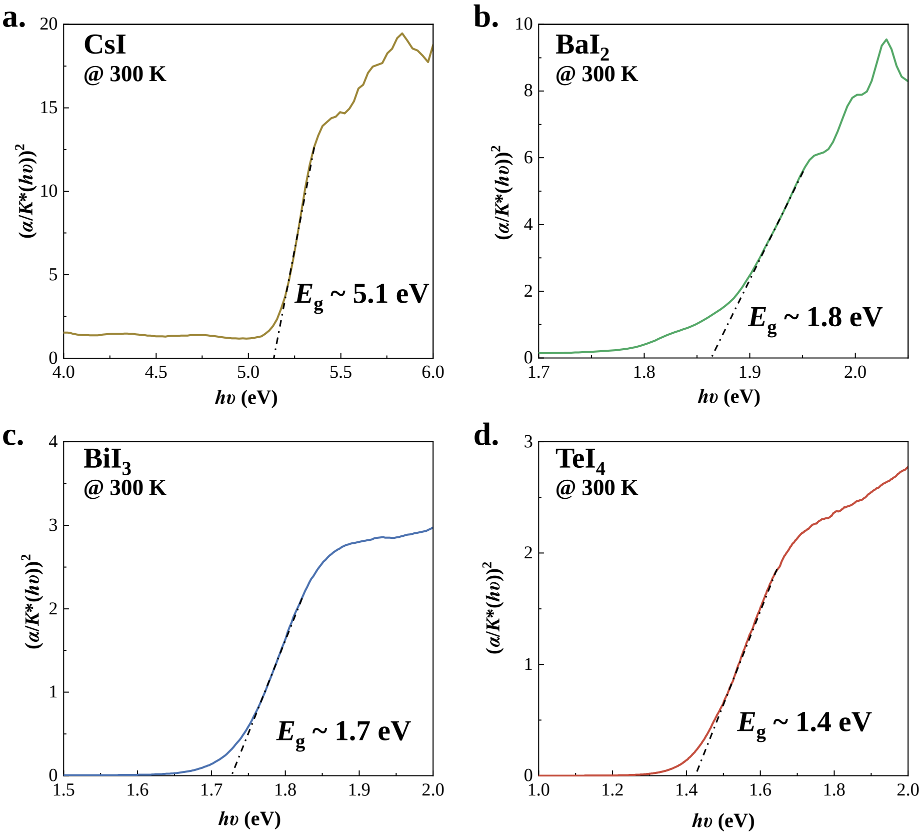

Figure S5. Optical band gap of poly-crystalline CsI (a), BaI2 (b), BiI3 (c) and TeI4 (d).

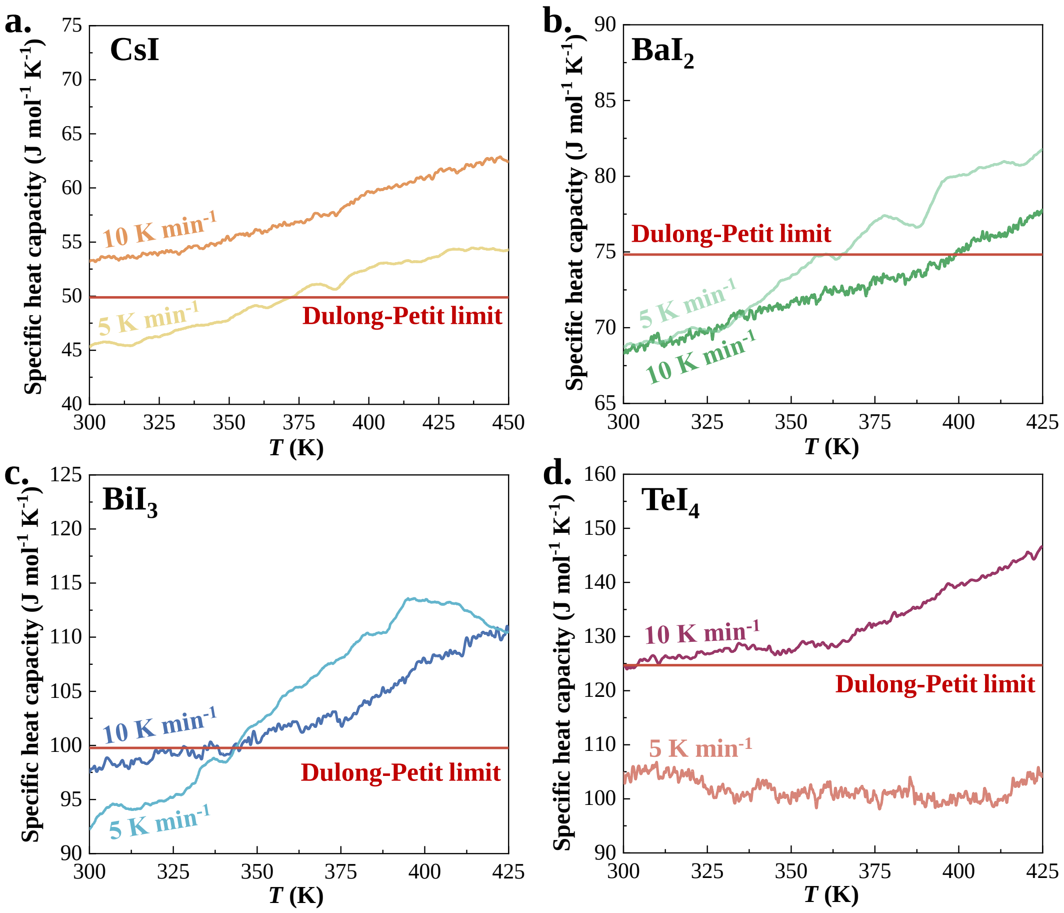

Figure S6. Specific heat of CsI (a), BaI_2 (b), BiI_3 (c), and TeI_4 (d), indicating the Dulong-Petit limit as an effective approximation (at least for T<425 K) by averaging all available data with different heating rates.

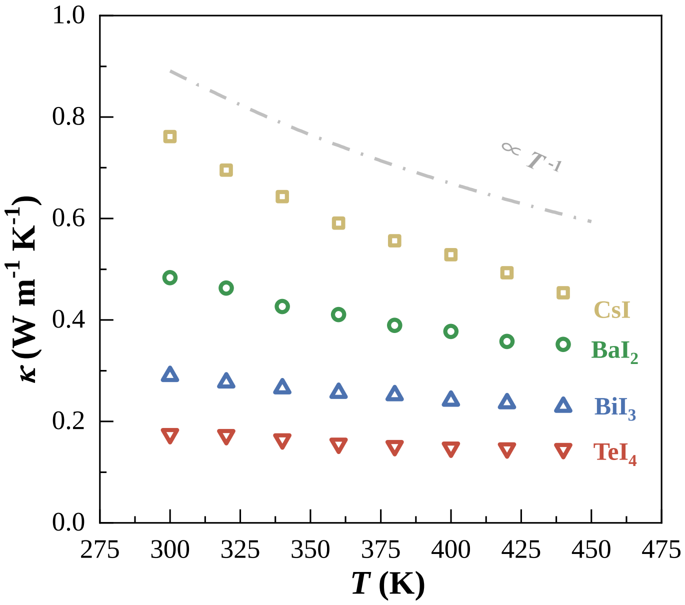

Figure S7. Temperature dependent total thermal conductivity (κ) for poly-crystalline CsI, BaI_2, BiI_3 and TeI_4.

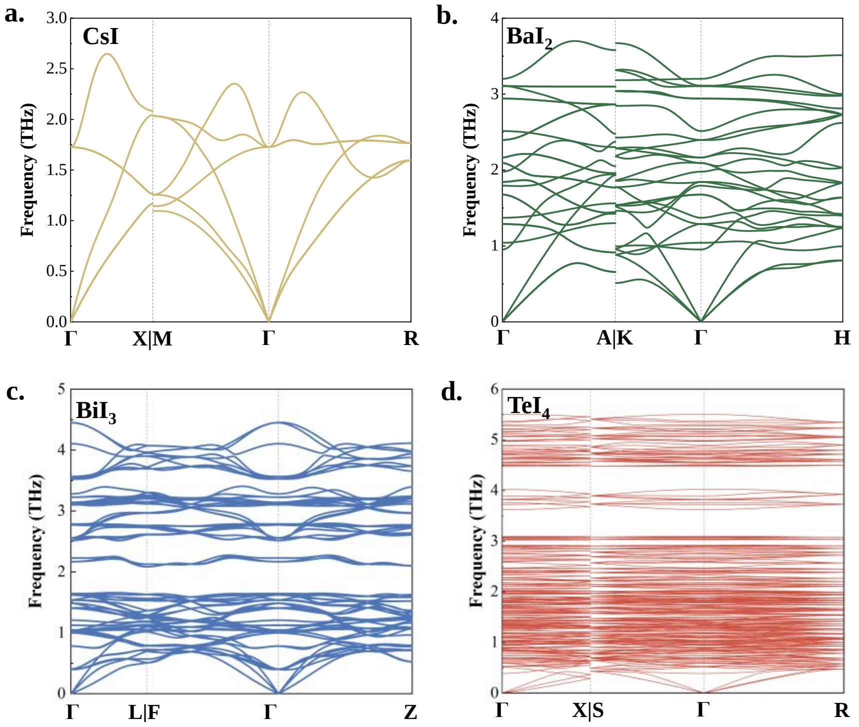

Figure S8. Calculated phonon spectra for CsI (a), BaI_2 (b), BiI_3 (c) and TeI_4 (d) based on finite-displacement method.

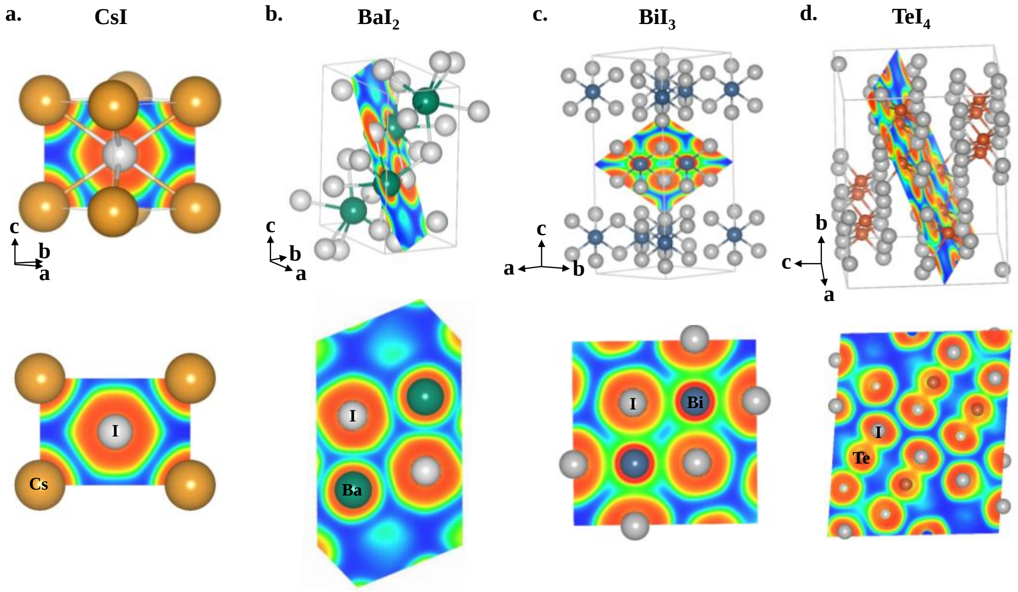

Figure S9. Calculated electron localization function for CsI (a), BaI_2 (b), BiI_3 (c) and TeI_4 (d).

Table S1. Crystallographic data for CsI, BaI_2, BiI_3 and TeI_4 at 300 K.

Materials	CsI	BaI_2	BiI_3	TeI_4
T, K	300	300	300	300
Molar mass, g mol^{-1}	260	391	590	635
a, Å	4.56670	9.142	7.516	13.6355
b, Å	4.56670	9.142	7.516	16.7985
c, Å	4.56670	5.173	20.718	14.6245
α, °	90	90	90	90
β, °	90	90	90	90
γ, °	90	120	120	90
V, Å^3	95.3	374.42	1013.57	3349.49
$V_{primitive}$, Å^3	95.3	374.42	337.86	3349.49
Z	1	3	6	16
D_{calcd}, g cm^{-3}	4.53	5.20	5.80	5.04
Number of atoms in primitive cell (n)	2	9	8	80
Average bond length, Å	3.96	3.48	3.09	2.93

Table S2. The hot-pressing parameters for CsI, BaI_2, BiI_3 and TeI_4.

Materials	Relative density (%)	Sintering temperature (K)	Sintering pressure (MPa)	Sintering time (min)
CsI	98	723	50	30
BaI_2	97	823	50	30
BiI_3	96	573	50	20
TeI_4	98	453	30	30

Table S3. Thermal Conductivity of CsI and TeI_4 along the a-, b-, and c-axes from AIMD simulations at 300 K.

κ (W m^{-1} K^{-1})	CsI	TeI_4
a-axis	1.696	0.146
b-axis	1.696	0.179
c-axis	1.696	0.156

Table S4. The physical parameters (Debye temperature θ_D, Shear modulus G, Bulk modulus B, Poisson ratio σ and Grüneisen constant γ) obtained from measured sound velocity at room temperature for CsI, BaI_2, BiI_3 and TeI_4.

Materials	Θ_D (K)	G (GPa)	B (GPa)	σ	γ
CsI	130	9.1	21.2	0.31	1.8
BaI_2	132	9.9	18.6	0.27	1.6
BiI_3	107	7.2	20.2	0.34	2.1
TeI_4	109	6.6	10.6	0.24	1.5

Table S5. Thermal conductivity comparison incorporates literature data. *n* represents the number of atoms per primitive cell, *v* denotes the sound velocity, and κ is the thermal conductivity.

Materials	n	v (m s^{-1})	κ(W m^{-1} K^{-1})	Materials	n	v (m s^{-1})	κ(W m^{-1} K^{-1})
LiF	2	4761[10]	14.2[10]	NaF	2	3684[10]	10.5[10]
KF	2	2840[10]	7.1[10]	RbF	2	2381[10]	2.3[10]
NaCl	2	2879[10]	6[11]	KCl	2	2395[10]	6.7[10]
RbCl	2	1851[10]	2.1[10]	AgCl	2	1632[10]	1.43[10]
CsCl	2	1765[10]	0.97[10]	LiBr	2	2302[10]	1.8[10]
NaBr	2	2124[10]	2.5[10]	KBr	2	1877[10]	3.8[10]
NaI	2	1691[10]	1.33[10]	CsBr	2	1549[10]	0.86[10]
γ-AgI	2	1271[12]	0.4[13]	MgO	2	7126[14]	60[15]
CaO	2	5427[16]	27[15]	SrO	2	3814[16]	12[15]
BaO	2	2726[16]	2.3[15]	MnO	2	3940[17]	10[18]
CoO	2	3956[17]	17[19]	CsI	2	1576	0.76
BaI_2	9	1533	0.48	BiI_3	8	1251	0.28
TeI_4	80	1271	0.17				

Supplementary references

[1] LaLonde, A. D., Ikeda, T., Snyder, G. J. Rapid consolidation of powdered materials by induction hot pressing [J]. *Rev. Sci. Instrum.*, 2011; **82**: 025104.

[2] Agne, M. T., Voorhees, P. W., Snyder, G. J. Phase transformation contributions to heat capacity and impact on thermal diffusivity, thermal conductivity, and thermoelectric performance [J]. *Adv. Mater.*, 2019; **31**: 1902980.

[3] G. Kresse, J. F. l. Efficient iterative schemes for ab initio total-energy calculations using a plane-wave basis set [J]. *Phys. Rev. B*, 1996; **54**: 11169-11186.

[4] Blöchl, P. E. Projector augmented-wave method [J]. *Phys. Rev. B*, 1994; **50**: 17953-17979.

[5] John P. Perdew, K. B., Matthias Ernzerhof. Generalized gradient approximation made simple [J]. *Phys. Rev. Lett.*, 1996; **77**: 3865-3868.

[6] Togo, A., Tanaka, I. First principles phonon calculations in materials science [J]. *Scr. Mater.*, 2015; **108**: 1-5.

[7] Han, S., Dai, S., Ma, J., et al. Strong phonon softening and avoided crossing in aliovalence-doped heavy-band thermoelectrics [J]. *Nat. Phys.*, 2023; **19**: 1649-1657.

[8] Eriksson, F., Fransson, E., Erhart, P. The hiphive package for the extraction of high-order force constants by machine learning [J]. *Adv. Theory Simul.*, 2019; **2**: 1800184.

[9] Li, W., Carrete, J., A. Katcho, N., et al. ShengBTE: A solver of the Boltzmann transport equation for phonons [J]. *Comput. Phys. Commun.*, 2014; **185**: 1747-1758.

[10] DB Sirdeshmukh, L. S., KG Subhadra. Alkali halides: A handbook of physical properties [M]. Springer Science & Business Media, 2001.

[11] B Håkansson, P. A. Thermal conductivity and heat capacity of solid NaCl and NaI under pressure [J]. *J. Phys. Chem. Solids*, 1986; **47**: 355-362.

[12] Shen, S.-G. Calculation of the elastic properties of semiconductors [J]. *J. Phys. Condens. Matter*, 1994; **6**: 8733.

[13] Wang, Y., Gan, Q., Hu, M., et al. Anharmonic lattice dynamics and the origin of intrinsic ultralow thermal conductivity in AgI materials [J]. *Phys. Rev. B*, 2023; **107**: 064308.

[14] Sumino, Y., Ohno, I., Goto, T., et al. Measurement of elastic constants and internal frictions on single-crystal MgO by rectangular parallelepiped resonance [J]. *J. Phys. Earth*, 1976; **24**: 263-273.

[15] Morelli, D. T., Slack, G. A. High lattice thermal conductivity solids [M]. New York, NY 10013, USA: Springer Science+Business Media, Inc., 2006.

[16] Z.P. Chang, E. K. G. Elastic properties of oxides in the NaCl-structure [J]. *J. Phys. Chem. Solids*, 1977; **38**: 1355-1362.

[17] Uchida, N., Saito, S. Elastic constants and acoustic absorption coefficients in MnO, CoO, and NiO single crystals at room temperature [J]. *J. Acoust. Soc. Am.*, 1972; **51**: 1602-1605.

[18] Slack, G. A., Newman, R. Thermal conductivity of MnO and NiO [J]. *Phys. Rev. Lett.*, 1958; **1**: 359-360.

[19] Saunders, F. B. L. a. N. H. The thermal conductivity of NiO and CoO at the Neel temperature [J]. *J. Phys. C: Solid State Phys.*, 1973; **6**: 2525.
